# Are Foods with Protein Claims Healthy? A Study of the Spanish Market

**DOI:** 10.3390/nu16244281

**Published:** 2024-12-11

**Authors:** Marta Beltrá, Fernando Borrás, Ana B. Ropero

**Affiliations:** 1 Institute of Bioengineering, Miguel Hernández University, 03202 Elche, Spain; beltra@umh.es; 2 Department of Statistics, Mathematics and Informatics, Miguel Hernández University, 03202 Elche, Spain; f.borras@umh.es

**Keywords:** protein claims, fortification, food database, nutrient composition, nutrition claims, nutrient profile/profiling model, nutritional quality, sweeteners, healthy foods

## Abstract

Background: Foods with protein claims (PCs) targeted at the general population are increasingly sought after by consumers because they think they are healthy. However, they may contain other nutrients that pose a health risk. Objectives: Therefore, the aim of this work was to carry out a comprehensive evaluation of foods with PC and compare them with those without these claims. Methods: The Spanish Food Database, BADALI, was used for this purpose. We studied 4325 processed foods of 12 different types. Thirteen percent had PCs and more than half of them were fortified with proteins (60.4%). Plant proteins were added more frequently than animal proteins. Protein values were higher in foods with PCs, particularly in those that were fortified. Differences in other nutrients were also observed depending on the food type. The healthiness of foods was assessed using the Pan American Health Organization Nutrient Profile Model and 90.8% of those bearing PCs were classified as “less healthy”. More than 50% were high in fat or high in sodium; around one in four were high in free sugar or saturated fat and one in five had sweeteners. Foods with PCs had 13.1% more “less healthy” items than those without PCs. The proportion of items high in fat or high in sodium were also larger and more of them had sweeteners. In contrast, the proportion of foods high in free sugar and high in saturated fat was lower among those with PCs. Conclusions: Therefore, the perception that foods with PCs are healthy is incorrect, and consuming them may pose an additional health risk.

## 1. Introduction

Protein and amino acid supplements, as well as protein-fortified foods have been on the market for some time in sports specialty shops [1,2]. In recent years, protein foods have been increasingly targeted to the general population. In fact, protein is among the best-selling functional ingredients in the U.S. at present and continues to rise [3]. According to data gathered by Innova Market Insights, there was a 26% compound annual growth rate in the number of food and beverage new-product launches making protein claims (PCs) in Europe in 2017–2022, and a 12% growth rate in Australasia [3,4]. A global survey in 2022 reported that 17% of consumers sought high-protein products [3]. This rise in popularity comes despite their association with higher prices [5,6].

Increasing the protein content of foods for the general population is not the result of the need for more protein intake. In fact, the majority of the population meets the recommendations in Western countries [7,8]. This growing interest in protein as functional ingredients may stem from consumer awareness of the important role these nutrients play in our bodies. In fact, a study conducted in Scotland and England reported that 69.8% of participants agreed that proteins are important for a healthy body, while 87% commented that they are important for muscles [9]. In addition, consumers seem to have a favorable opinion of proteins as ingredients, which are frequently used to fortify foods [10].

The extra protein may influence consumers’ perception of these foods and may alter their perceived healthiness. In fact, the general feeling of being “healthy” was one of the most important characteristics of protein-fortified foods for consumers in the U.S. and in four European countries [5,11]. A study of consumers who tried yogurts concluded that “the dimension healthy food was associated with yogurt fortified with proteins” [12].

In laboratory-controlled experiments, consumers are told which foods are fortified. However, in real, uncontrolled settings, consumers can tell by reading the ingredient list and comparing the protein value to conventional foods. Nevertheless, this is impractical for the average consumer and, instead, they choose based on the presence of nutrition claims (NCs). According to regulation, fortification is not required for the use of NCs about proteins [13]. Therefore, both fortified and unfortified foods can carry these PCs as long as they meet the criteria [13]. As a result, consumers seeking protein fortified foods tend to choose foods with PCs.

PCs can alter consumers’ perception of food. In a recent study, breakfast cereals that have the word “protein” on the front of the package were perceived as providing “greater health benefits, such as being more likely to help them build muscle, stay healthy, and live longer” [14]. Similarly, a study conducted in the U.S. concluded that “protein” bars were perceived as healthier and thought to have more protein [15]. Additional evidence comes from a study with Polish consumers, who identified dairy products with the high protein claim as having a high nutritional value [16]. Rramani et al. also observed that the “protein-rich” claim increased the expected and perceived healthiness of milk-mix drinks and reduced reward-related responses in the brain during tasting [17].

Despite this favorable perception, foods with PCs may contain nutrients that negatively affect health. Consumers may overlook these threats if they only pay attention to the PCs. In fact, a study on breakfast cereals reported that consumers did not realize the higher sugar, sodium and calorie content in those with PCs [14]. Carrying PCs may even overshadow the presence of warning labels about other nutrients, such as sugar and calorie content [15].

As we have seen, foods with PCs are increasingly sought after by consumers because they think they are healthy. In fact, consumers may believe that they are choosing what is best for their health. However, they may not realize that these foods may contain other nutrients that pose a health risk. Therefore, we considered it critical to evaluate the nutritional quality of foods with PCs. For this purpose, we studied foods available in the Spanish market and included in the Food Database, BADALI. The prevalence of foods carrying PCs, along with their nutritional composition and quality, was studied. They were also compared to foods without PCs. In addition, the proportion of protein fortified items was analyzed, as well as the types of proteins added.

## 2. Materials and Methods

### 2.1. BADALI Database of Foods Available in the Spanish Market

The data used in this work come from the Food Database, BADALI, developed by the authors at Miguel Hernández University [18]. The characteristics of the BADALI database can be found elsewhere [19]. The inclusion criteria for foods in the BADALI database were as follows: (1) foods sold in any Spanish supermarket and (2) foods with the nutrition declaration. Both manufacturers’ and supermarkets’ websites were used to collect data following the methodology described in Ropero et al., 2023 [19]. The information was extracted; inconsistent data were not used for further analysis and duplicates (different pack sizes) were removed. When the energy content was not provided, it was calculated by using the following coefficients: 4 kcal/g for protein and carbohydrate; 9 kcal/g for total fat; 2.4 kcal/g for polyols and 2 kcal/g for fiber [20]. Salt content, but not sodium, is mandatory in the nutrition declaration according to the European Regulation (EU) 1169/2011 [20]. Therefore, the sodium content was calculated by dividing the salt values by 2.5. For the present study, data were gathered from June 2022 to March 2024 and the items were classified into twelve distinct food types (Supplementary Table S1).

### 2.2. Protein Claims (PCs) and Fortification

The main image of the food displayed in the manufacturers’ or the supermarkets’ website was checked for protein claims (PCs). Foods with unreadable images were excluded. An item was listed as having a PC if the word “protein” was displayed in the main image. In the present work, it was estimated that nutrition claims on proteins, as defined in the European Regulation (EC) No 1924/2006 (“rich/high in protein”, “source of protein” or similar), accounted for around 90% of all PCs [13]. The remaining claims were of the “19 g of protein” type, which accounted for the rest. These are not formal nutrition claims [13].

Protein fortification was defined as the addition of specific proteins according to the presence of any of the following items in the ingredient list: (1) one that has the word “protein”; (2) any amino acid or (3) gluten, collagen, whey, gelatine, albumin, egg white or calcium caseinate. The addition of ultra-filtered, concentrated or powdered milk, cereal or legume flours was not considered as protein fortification because they also provide other nutrients. Gluten added to bread was not considered protein fortification because it is usually added to improve structural properties.

### 2.3. Nutrient Composition Analysis

Nutrient composition analysis was performed as previously reported [19]. Briefly, to consider that statistically significant divergences were also nutritionally relevant, 30% changes in median values were required, except for sodium (25%) [13,19] (values with **). Only food types with at least 30 items/conditions were analyzed when comparing foods with and without PCs. The minimum sample size was reduced to 8 items/condition for comparisons between fortified and unfortified foods (with PCs).

### 2.4. Evaluation of Food Healthiness

The Nutrient Profile Model established by the Pan American Health Organization and the World Health Organization—Regional Office in the Americas (PAHO-NPM) was used to classify food as “healthy” or “less healthy” as in previous works [19,21,22,23]. The PAHO-NPM criteria are not to be applied to food without added fat, salt, sweeteners or free sugar. Therefore, these were considered “healthy” foods. On the contrary, items were classified as “less healthy” when reaching or exceeding any threshold for critical nutrients (sodium, free sugar, total fat, saturated fat, trans fat) or contained any low- or no-calorie sweeteners (LNCS) [23]. The criteria for trans-fat could not be applied because none of the products provided information on their trans-fat content. Only foods with data for the rest of the nutrients were included in the statistics for the “less healthy” parameter. Thresholds were as follows: (1) ≥1 mg sodium/kcal, (2) ≥10% of total energy from free sugars, (3) ≥30% of total energy from total fat and (4) ≥10% of total energy from saturated fat [23]. Polyols were not registered as sweeteners when used as humectants, bulking agents or with any other function. Free sugar content was estimated following WHO, 2015 and Swan et al., 2018 [24,25] as follows: (1) All sugar present in milk/yogurt/dairy dessert substitutes and fruit drinks was considered free, except for added milk; (2) Cereal-based products (cereal bars, biscuits, breakfast cereals) with no added sugar have no free sugar; (3) 2 g sugar/100 g was subtracted from total sugar in biscuits, cereal bars and breakfast cereals because this is the natural sugar content in the most commonly used grains [26]; (4) 5 g sugar/100 g was subtracted from total sugar content for milk/dairy drinks and yogurts/fermented milk because this is the maximum lactose content in milk [26]; (5) Free sugar in plant-based meat analogues was estimated according to the ingredient declaration; (6) The sugar naturally present in nuts was not considered free; (7) Sugar in fruit and dry fruit was considered free when added in puree or paste (in bars and yogurts) and (8) Although the sugar content was not available in some products, free sugars could be estimated based on the ingredient declaration. Free sugar content of some bars was impossible to estimate because the proportion of dry fruit in paste, puree or in any other form was not provided.

### 2.5. Statistics

The Kruskal–Wallis H test, also known as the “one-way ANOVA on ranks,” is a nonparametric statistical method using rank-based analysis. This test is utilized to ascertain statistically significant disparities among two or more categories of an independent variable with respect to a continuous or ordinal dependent variable. While the nonparametric ANOVA does not require the assumption of normality in random error distribution, it does require the independence of random errors. To evaluate whether different columns (or rows) of data within a table originate from an identical population, the chi-square test of homogeneity was used. This test determines if the observed differences are due only to sampling error. For all statistical analyses, the significance threshold was set at *p* < 0.05.

The statistical analysis of the application data in this work was performed using Microsoft Excel and Google Colab with Jupyter Notebooks, along with the libraries scikit-learn 0.22.2. post 1, Pandas v0.25.3 and Matplotlib Python v3.2.0. Violin plots were created using the R version 4.4.1 [27].

## 3. Results

### 3.1. Description of the Sample and Protein Fortification

This study included 4325 processed foods and classified into twelve specific food types (Table 1 and Supplementary Table S1). As many as 561 items carried protein claims (PC) (13%) (see Section 2.2. for the definition of PC) and the prevalence varied across food types (Table 1). Plant-based meat analogues had the highest proportion of foods with PCs (68.2%), followed by bars (35.3%) and yogurt/dairy dessert substitutes (21.3%). However, none of the biscuits or fruit drinks had PCs.

Protein fortification was analyzed next. Up to 60.4% of the items were fortified, while the rate was around 90% for bars and plant-based meat analogues (90.5% and 89.6%, respectively) (Table 1). On the contrary, while only a few milk substitutes and yogurt/dairy dessert substitutes were fortified (7.9% and 3.3%, respectively). More than half of fortified foods added only one protein ingredient (55.2%), while 23.3% added two and 18.6% added three (Supplementary Figure S1). The list of these ingredients included 23 items (Supplemental Table S2). Plant proteins were more frequently added than animal proteins (41.7% and 25.9% of foods with PCs). Gluten was the leader, followed by milk and soy proteins (18.9%, 18.7% and 18.5%, respectively) (Supplementary Table S2). The complete distribution of protein ingredients by food type is presented in Supplementary Table S3.

### 3.2. Protein Content

Statistically significant increases in protein content were observed in the seven analyzed food types (≥30 items/condition), and five of these were also nutritionally relevant (≥30% changes) (Table 2). Strikingly, the median protein values in milk substitutes and bars with PCs were four times higher, while plant-based meat analogues with PCs had more than twice as much protein (Table 2). The higher protein content in milk/yogurt/dairy dessert substitutes with PCs was because soy was the main ingredient in more than 90% of the items (92.1% in milk substitutes and 93.3% in yogurt/dairy dessert substitutes). Soy was much less present in those without PCs (10.7% milk substitutes and 42.3% in yogurt/dairy dessert substitutes).

Differences in protein content may be greatly influenced by fortification. In fact, fortified foods with PCs had a higher protein content in four of the five food types analyzed (≥8 items/condition) (Supplementary Table S4 and Figure S2). The differences in protein content were more than double for bars and bread (133% and 158% more, respectively), while there were none in milk/dairy drinks.

### 3.3. Nutrient Composition

The rest of the nutrients were also compared and many statistically significant differences were obtained, although only those that were nutritionally relevant will be commented on (see Material and Methods; highlighted with ** in Table 2). Relevant changes were observed in carbohydrates, sugar, total and saturated fat, while no important differences occurred in energy, fiber or sodium.

Bars with PCs presented a significant reduction in carbohydrates (49% less), mainly due to the 82% reduction in sugar content (Table 2). They also had more saturated fat (48% more). Milk/dairy drinks with PCs contained less total and saturated fat, while yogurts/fermented milk also showed less carbohydrates and sugar. Although the same results were observed in milk substitutes regarding carbohydrates and sugar, they had more total fat. As for plant-based meat analogues, the carbohydrate content of those with PCs was lower with no other relevant change. Finally, bread did not present any nutritionally relevant difference.

### 3.4. Nutritional Quality

Food healthiness was evaluated using the Nutrient Profile Model developed by the Pan American Health Organization and the World Health Organization—Regional Office in the Americas (PAHO-NPM) [23]. The results show that 90.8% of foods with PCs were classified as “less healthy” and more than 50% were high in fat or high in sodium (52.3% and 53.7%, respectively) (Table 3). In addition, around one in four were high in free sugar or saturated fat (24.5% and 26.7%, respectively) and one in five had sweeteners (19.3%).

When both conditions were compared, foods with PCs presented 17% more items that were “less healthy” (77.7% vs. 90.8%) (Table 3). The percentage of foods high in fat or high in sodium were also larger (74% and 79% more, respectively), and more of them had sweeteners (164% more). In contrast, the proportion of foods high in free sugar and high in saturated fat was lower among those with PCs (50% and 16% less, respectively).

**Table 2:** Energy and nutrient density of specific food types. Values in 100 g or 100 mL.

Food Types	Protein Claims (PCs)	Energy (kcal)			Protein (g)			Carbohydrates (g)			Sugar (g)		
		*n*	Median (IR)	*p*-Value	*n*	Median (IR)	*p*-Value	*n*	Median (IR)	*p*-Value	*n*	Median (IR)	*p*-Value
Bars	No	174	430 (389; 470)	<0.01 *	174	7.5 (5.7; 11.9)	<0.001 **	174	59 (48; 66)	<0.001 **	174	28.9 (21.5; 35.7)	<0.001 **
	Yes	95	381 (358; 485)		95	30 (23; 36)		95	30 (23; 39)		95	5.2 (3.2; 20.3)	
Bread	No	309	258 (242; 276)	0.902	309	9 (5.4; 9.9)	<0.001 *	309	45 (41; 49)	<0.001 *	300	3.8 (2.4; 5.1)	<0.05 *
	Yes	36	260 (246; 274)		36	11.1 (9.7; 16.7)		36	39.5 (26; 45)		36	3.5 (1.9; 3.8)	
Milk/dairy drinks	No	371	49 (44; 63)	0.114	371	3.1(3; 3.2)	<0.001 **	371	4.8 (4.7; 5.1)	0.978	368	4.8 (4.7; 5)	0.597
	Yes	31	54 (46; 65)		31	6 (5; 9.4)		31	4.8 (4.7; 5.1)		31	4.8 (4.6; 5.1)	
Milk substitutes	No	297	48 (39; 59)	0.066	298	0.8 (0.5; 1.2)	<0.001 **	297	7.8 (3.6; 10)	<0.001 **	298	4.8 (2.2; 6)	<0.001 **
	Yes	38	42 (34; 52)		38	3.4 (3; 3.8)		38	2.5 (1.3; 4.5)		38	1.8 (0.6; 3.8)	
Plant-based meat analogues	No	87	197 (176; 232)	<0.05 *	90	7.2 (5; 12)	<0.001 **	87	15 (6.3; 21.2)	<0.05 **	90	2 (1.1; 2.9)	<0.05 *
	Yes	193	218 (185; 244)		193	16 (11.4; 19)		193	10.2 (5.8; 17)		193	1.5 (0.8; 2.6)	
Yogurt/dairy dessert substitutes	No	111	87 (75; 109)	0.090	111	2.1 (1; 3.7)	<0.001 **	111	11 (5.9; 14.9)	0.075	111	8 (2.5; 11)	0.726
	Yes	30	82 (75; 89)		30	3.8 (3.8; 4.6)		30	9.9 (4.1; 12)		30	9.5 (3; 11.3)	
Yogurts/fermented milk	No	736	75 (56; 97)	<0.001 *	736	3.5 (3; 4.1)	<0.001 *	736	10.8 (5.5; 12.2)	<0.001 **	732	10.6 (5.1; 12)	<0.001 **
	Yes	95	58 (45; 75)		95	4.2 (3.2; 7.5)		95	5.4 (4.7; 11)		95	5.2 (4.4; 10.8)	
**Food Type**	**Protein Claims (PCs)**	**Total Fat (g)**			**Saturated Fat (g)**			**Fiber (g)**			**Sodium (mg)**		
		***n***	**Median (IR)**	***p*-Value**	***n***	**Median (IR)**	***p*-Value**	***n***	**Median (IR)**	***p*-Value**	***n***	**Median (IR)**	***p*-Value**
Bars	No	174	15.7 (10.9; 23)	0.662	172	4.6 (2.6; 8.1)	<0.001 **	146	6.9 (5.3; 8.7)	0.895	172	94 (24; 200)	0.272
	Yes	95	14.2 (11; 21.7)		95	6.8 (4.6; 9.7)		91	6.3 (4.3; 14)		95	104 (72; 166)	
Bread	No	308	4 (2.7; 5.4)	<0.05 *	294	0.7 (0.5; 1)	<0.05 *	254	ND	ND	299	440 (400; 520)	0.749
	Yes	36	4.9 (2.9; 9)		36	0.8 (0.6; 1.1)		26	ND		36	440 (400; 490)	
Milk/dairy drinks	No	371	1.6 (0.6; 2.4)	<0.001 **	366	1 (0.4; 1.3)	<0.001 **	54	ND	ND	366	52 (48; 52)	<0.001 *
	Yes	31	0.5 (0.3; 1.5)		31	0.3 (0.2; 0.9)		6	ND		31	56 (52; 68)	
Milk substitutes	No	298	1.4 (1; 2.1)	<0.001 **	298	0.2 (0.1; 0.4)	<0.01 **	198	0.5 (0.3; 0.8)	0.251	293	40 (28; 40)	<0.05 *
	Yes	38	1.9 (1.8; 2.1)		38	0.3 (0.3; 0.4)		33	0.6 (0.5; 0.8)		38	40 (36; 60)	
Plant-based meat analogues	No	90	10.6 (6.3; 15)	0.825	90	1.3 (0.8; 1.8)	0.242	60	4 (3;5.6)	0421	90	580 (440; 800)	<0.01 *
	Yes	192	10.8 (7.1; 15)		192	1.3 (1; 1.9)		161	3.9 (2.7; 5.5)		192	520 (440; 600)	
Yogurt/dairy dessert substitutes	No	111	3 (2.1; 5.1)	0.111	111	0.5 (0.4; 2.6)	<0.01 **	39	ND	ND	111	40 (20; 44)	0.484
	Yes	30	2.2 (2.1; 3)		29	0.3 (0.3; 0.6)		21	ND		30	40 (29; 40)	
Yogurts/fermented milk	No	733	2.3 (1; 3.8)	<0.001 **	731	1.6 (0.3; 2.5)	<0.001 **	840	ND	ND	736	44 (40; 52)	0.076
	Yes	95	0.4 (0.1; 1.5)		95	0.2 (0.1; 1.1)		9	ND		95	48 (40; 60)	

*n*: Foods with data. IR: interquartile range. * Statistically significant differences according to *p* < 0.05. ** Statistically significant differences that are also nutritionally relevant (at least 30% changes, 25% for sodium, see Section 2.3). ND = not determined because of <30 foods/condition.

**Table 3:** Classification of foods as high in critical nutrients according to the PAHO-NPM, by food type [23].

Food Types	Protein Claims (PCs)	Less Healthy			High Fat			High Free Sugar			High Saturated Fat			High Sodium			Sweeteners (LNCS)		
		*n*	No (%)	*p*-Value	*n*	No (%)	*p*-Value	*n*	No (%)	*p*-Value	*n*	No (%)	*p*-Value	*n*	No (%)	*p*-Value	*n*	No (%)	*p*-Value
Bars	No	151	150 (99.3)	0.062	172	100 (58.1)	0.79	152	136 (89.5)	<0.001 *	172	79 (45.9)	<0.001 *	170	2 (1.2)	1	171	7 (4.1)	<0.001 *
	Yes	94	89 (94.7)		94	57 (60.6)		94	40 (42.6)		94	79 (84)		94	1 (1.1)		94	32 (34)	
Bread	No	282	269 (95.4)	0.385	306	14 (4.6)	<0.001 *	306	15 (4.9)	0.353	292	5 (1.7)	0.944	297	281 (94.6)	0.310	307	0 (0)	1
	Yes	36	36 (100)		36	9 (25)		36	0 (0)		36	0 (0)		36	36 (100)		36	0 (0)	
Milk/dairy drinks	No	371	81 (21.8)	<0.001 *	371	7 (1.9)	0.955	371	68 (18.3)	<0.05 *	371	51 (13.8)	1	371	35 (9.4)	<0.05 *	371	8 (2.2)	<0.001 *
	Yes	31	19 (61.3)		31	0 (0)		31	0 (0)		31	4 (12.9)		31	7 (22.6)		31	18 (58.1)	
Milk substitutes	No	292	285 (97.6)	0.175	297	132 (44.4)	<0.001 *	297	234 (78.8)	0.051	297	41 (13.8)	0.09	292	83 (28.4)	0.123	298	6 (2)	0.114
	Yes	38	35 (92.1)		38	30 (78.9)		38	24 (63.2)		38	1 (2.6)		38	16 (42.1)		38	3 (7.9)	
Plant-based meat analogues	No	86	86 (100)	0.219	86	66 (76.7)	0.418	86	0 (0)	0.853	86	12 (14)	1	86	85 (98.8)	0.346	89	0 (0)	1
	Yes	188	182 (96.8)		192	157 (81.8)		191	2 (1)		192	27 (14.1)		192	184 (95.8)		193	0 (0)	
Yogurt/dairy dessert substitutes	No	111	103 (92.8)	0.485	111	57 (51.4)	0.122	111	87 (78.4)	1	111	39 (35.1)	<0.05 *	111	8 (7.2)	1	111	0 (0)	1
	Yes	30	26 (86.7)		30	10 (33.3)		30	23 (76.7)		30	3 (10)		30	2 (6.7)		30	0 (0)	
Yogurts/fermented milk	No	705	579 (82.1)	1	707	238 (33.7)	<0.001 *	709	451 (63.6)	<0.001 *	705	418 (59.3)	<0.001 *	709	117 (16.5)	<0.001 *	709	129 (18.2)	<0.001 *
	Yes	95	78 (82.1)		95	7 (7.4)		95	37 (38.9)		95	24 (25.3)		95	31 (32.6)		95	47 (49.5)	
Total	No	1998	1553 (77.7)	<0.001 *	2050	614 (30)	<0.001 *	2032	991 (48.8)	<0.001 *	2034	645 (31.7)	<0.05 *	2036	611 (30)	<0.001 *	2056	150 (7.3)	<0.001 *
	Yes	512	465 (90.8)		516	270 (52.3)		515	126 (24.5)		516	138 (26.7)		516	277 (53.7)		517	100 (19.3)	

*n*: foods with data; No: number of foods meeting each condition; %: percentage within the food type. *: Statistically significant differences according to *p* < 0.05. Thresholds used to consider foods as high in critical nutrients are: ≥30% of total energy from total fat, ≥10% of total energy from free sugars, ≥10% of total energy from saturated fat, ≥1 mg sodium/kcal (PAHO, 2016 [23]). LNCS: low- and no-calorie sweeteners.

When food types were studied individually, many differences were observed (Table 3). The most striking was the greater proportion of foods with sweeteners in milk/dairy drinks, bars and yogurts/fermented milk with PCs (26, 8 and 2 times more, respectively). More yogurts/fermented milk items with PCs were high in sodium, while lower proportion were high in fat, in free sugar and in saturated fat (78% more, 39% and 57% less, respectively). As for milk/dairy drinks, almost three times as many foods with PCs were “less healthy” (61.3% vs. 21.8%) and more than double were high in sodium (140% more). Eighty-three percent more bars were high in saturated fat among those with PCs (84% vs. 45.9%), while 52% less were high in free sugar (42.6% vs. 89.5%). Bread presented five times as many items that were high in fat (25% vs. 4.6%). Milk substitutes only differed in the proportion of foods that were high in fat, which was 78% higher in those with PCs (78.9% vs. 44.4%). Results were not different in plant-based meat analogues and yogurt/dessert dairy substitutes with PCs had less proportion of items high in saturated fat (10% vs. 35.1%).

## 4. Discussion

To our knowledge, this is the first comprehensive study on the nutritional quality of foods with protein claims (PCs). Around one in seven foods had PCs and less than two-thirds of them were fortified with proteins, primarily from plant sources. The nutritional comparison between foods with and without PCs showed striking differences in protein content, as well as in sugar, total and saturated fat. The overall nutritional quality of foods with PCs was worse than that of those without these claims. Nine out of ten items were classified as “less healthy”.

Proteins have never been a popular subject of nutrition claims (NCs) for food promotion [28,29,30,31]. Previous works have reported rates of 0.5% and 1.7% in several European countries (calculated from data in references [29,30], respectively). Our own work published in 2020 reported nearly 9% [31]. The 5% prevalence of PCs in breakfast cereals reported here is similar to the 7.2% obtained in Ireland, while it is much lower than the 23% in Australia (calculated from data in references [29,32]). Similar rates were also obtained in bread compared to Ireland (10.4% here and 11.6% in Ireland), although this also included bakery products (value calculated from data in reference [29]). The prevalence in yogurts and yogurt drinks was much higher in Ireland (32.1% vs. 11.4%) (value calculated from data in reference [29]).

### 4.1. Nutritional Quality of Foods with PCs

As may be expected, our findings show that foods with PCs contain more proteins than those without these claims. However, only two thirds of them were fortified and had more proteins than the unfortified foods (both with PCs). Therefore, when looking for foods with extra protein, one should compare their protein values. Nevertheless, this is not feasible for the average consumer and they choose based on the PCs, which do not guarantee a higher protein content.

According to our data, foods with PCs cannot be considered healthy because nine out of ten are classified as “less healthy”. Half of the items with PCs were high in sodium. Excessive sodium intake is the leading dietary risk factor for mortality in the world [33]. The primary health effect associated with high-sodium diets is increased blood pressure, which raises the risk of cardiovascular diseases [33]. Both globally and in Spain, the mean sodium intake in adults is approximately twice the recommended levels [34]. It is estimated that approximately three quarters of the total sodium intake in Europe and Northern American countries comes from salt added to processed foods [35]. Therefore, half of the foods with PCs should be reduced to lower the risk of noncommunicable diseases associated with excessive sodium intake.

One in four foods with PCs was classified as high in free sugar. A high free sugar intake is associated with poor dietary quality, obesity and risk of noncommunicable diseases [25]. In addition, they may contribute to a positive energy balance while reducing the intake of foods with higher nutritional quality [25]. Therefore, 25% of foods with PCs may contribute to the already high intake of free sugar in Spain and other European countries and its health consequences [36].

The lower proportion of foods high in free sugar among those with PCs may lead consumers to believe they are healthy. However, they use sweeteners more frequently. Research carried out over the past few decades has resulted in the 2023 report issued by WHO [37]. In the report, WHO “suggests that non-sugar sweeteners (polyols excluded) not be used as a means of achieving weight control or reducing the risk of noncommunicable diseases” [37]. In 2016, the Pan American Health Organization had warned that “habitual use of sweet flavors (sugar-based or not) promotes the intake of sweet food and drinks, including those that contain sugars” [23].

One in four foods with PCs were high in saturated fat as well as 61% bars. According to the evidence, lowering saturated fat intake reduces LDL-cholesterol and cardiovascular disease risk, and may also be associated with a reduced risk of all-cause mortality and coronary heart disease [38]. The Spanish population exceeds the recommended daily intake of saturated fats [39]. Therefore, the consumption of these foods may contribute to the already high burden of cardiovascular disease and increase the risk of mortality.

To our knowledge, only one study has been published that compares the nutritional quality of foods with and without PCs [40]. Authors analyzed more than 15,000 foods in the Canadian market and found that those with PCs had better nutritional quality (the nutrient composition was not compared) [40]. However, our data show otherwise. The possibility that the nutrient profile of foods with PCs has changed in the six years between the two studies cannot be ruled out, particularly because they have only recently been in the spotlight. Nonetheless, previous studies have shown that foods with nutritional and/or health claims tend to have better nutritional quality than those without such claims [40,41,42,43]. However, the results largely depend on the criteria used to assess the nutritional quality [42].

Consumers attribute healthy properties to foods with PCs [14,15,16,17]. According to our data, this perception is incorrect because only one in ten was healthy. Therefore, consumers may not be aware of the high health risk posed by other nutrients present in foods with PCs. In fact, an interesting study reported that consumers did not realize the higher sugar, sodium and calorie content in breakfast cereals with PCs [14]. The presence of PCs even has the potential to counteract the traffic light warning label on sugar and calorie content displayed in terms of perceived healthfulness [15].

### 4.2. Extra Proteins in the Diet

Physical activity increases the demand for protein intake and fortified foods may be used to guarantee the extra protein required. In fact, the International Society of Sports Nutrition Position Stand on protein and exercise acknowledged that “while it is possible for physically active individuals to obtain their daily protein requirements through the consumption of whole foods, supplementation is a practical way of ensuring intake of adequate protein quality and quantity, while minimizing caloric intake, particularly for athletes who typically complete high volumes of training” [44]. However, the consumption of fortified foods must be supervised by expert nutritionists.

The majority of the population meets the current recommended protein intake in Western countries, including Spain [7,8]. However, some authors propose increasing the protein intake for methodological and health-related reasons (healthy aging, appetite regulation, weight management) [45,46]. Foods with extra protein may be a viable option for increasing this intake, although potential negative effects should not be overlooked.

The increase in protein intake may be particularly important for the elderly. It is estimated that between 16% and 70% of elderly in residential care centers have protein–energy malnutrition [47]. Age-related anorexia is held responsible for most of the nutritional decline in residents [48,49]. The consequences of anorexia of the aging include weight loss, frailty, sarcopenia, malnutrition and impaired mobility [48,49]. It has been estimated that the prevalence of sarcopenia is 9.9–40.4% [50]. A recent systemic review and meta-analysis of observational studies concluded that a high protein consumption was associated with a significantly lower risk of frailty aged 60 and older [51]. Observational and interventional studies report benefits of higher protein intakes in elderly for improving body composition, preserving strength, general health and physical function [46]. Further increasing the protein intake in the elderly can be difficult because they already struggle to reach the current RDA. In fact, a study performed in five European countries (Spain, The Netherlands, UK, Finland and Poland) reported that 37.7% of adults aged 65 years and older had a high probability of inadequate protein intake [52]. The ANIBES study reported that 17% of men and 12.6% of women between 65 and 75 years old in Spain did not meet the protein recommendations [8]. Therefore, protein foods may be beneficial for this subpopulation, particularly for those in residential care centers.

The use of foods with extra protein as a means of increasing dietary protein intake has three major disadvantages. One is that these foods only provide extra protein, while natural protein foods (pulses, fish, meat) also provide other equally important nutrients (carbohydrates, fiber, vitamins, minerals). In fact, as the protein content increases, the presence of the rest of the nutrients may diminish. Another disadvantage is that, according to our data, plant proteins are preferentially used, and their quality is lower than that of animal proteins [53]. The third and most important of all is that only one in ten of these foods is healthy because they are high in critical nutrients negatively associated with health. Therefore, it is unacceptable for an increase in protein intake to be accompanied by a higher health risk.

Finally, this work has some strengths. It is the first comprehensive study of the nutritional quality of foods with PCs. It is also the first publication with a complete comparison of foods with and without PCs (nutrient composition and nutritional quality). Furthermore, the sample for each food type is large. This study has also some limitations that have already been reported in previous publications by the group [19]. One specific limitation should be added to this. We can only guess at the true intent of the manufacturer when adding ingredients, which may lead to underestimating or overestimating the fortification rate.

## 5. Conclusions

The analysis of foods with PCs reported in the present work suggests that they can be used to increase the protein intake, as suggested by some authors, and particularly in the elderly. However, foods with PCs are unhealthy, and their consumption poses an additional risk to our health that should never be justified by the presence of extra protein. This is particularly serious because consumers are often unaware of this risk and instead perceive these foods as healthy. Therefore, the presence of PCs on the packaging should never prevent consumers from checking the nutrition declaration and the ingredient list to make healthy choices.

## Figures and Tables

**Table 1:** Items included in this study, presence of protein claims (PCs) and fortification rates.

Food Types	Total (%) *	No Foods with PCs	
		Total (%)	Fortified (%) *
Bars	269	95 (35.3)	86 (90.5)
Biscuits	637	0 (0)	0 (0)
Bread	345	36 (10.4)	8 (22.2)
Breakfast cereals	424	21 (5)	6 (28.6)
Cereal cakes/crackers	181	9 (5)	1 (11.1)
Fruit drinks	251	0 (0)	0 (0)
Milk/dairy drinks	402	31 (7.7)	18 (58.1)
Milk substitutes	336	38 (11.3)	3 (7.9)
Plant-based meat analogues	283	193 (68.2)	173 (89.6)
Toasted bread	225	13 (5.8)	4 (30.8)
Yogurt/dairy dessert substitutes	141	30 (21.3)	1 (3.3)
Yogurts/fermented milk	831	95 (11.4)	39 (41.1)
Total	4325	561 (13)	339 (60.4)

No: number of foods; PCs: protein claims. %: percentage of the food sample, total or by food group; *: percentage of the sample with PCs, total or by food type.

## Data Availability

Data used in this work can be found at https://badali.umh.es (accessed on 6 November 2024).

## Supplementary Materials

The following supporting information can be downloaded at: https://www.mdpi.com/article/10.3390/nu16244281/s1, Table S1: Description of the food types included in the study, by food type; Figure S1: Presence of protein specific ingredients per food; Table S2: Protein ingredients added to fortified foods with protein claims (PC); Table S3: Number of foods with PC and protein ingredients added, by food type and ingredient; Table S4: Protein content in foods with protein claims (PC); Figure S2: Protein content in foods with protein claims (PC).
