# Supplementary material for: Are Foods with Protein Claims Healthy? A Study of the Spanish Market
Source: Nutrients. 2024 Dec 11;16(24):4281. doi: 10.3390/nu16244281 (PMC11679597; doi:10.3390/nu16244281)
Supplement: Supplementary file 1 [file nutrients-16-04281-s001.zip › nutrients-3331201-supplementary.pdf]

Table S1. Description of the items included in the study, by food type.

Food Types	Foods
Bars	Bars made of cereals, legumes, dry fruit or nuts with or without added ingredients
Biscuits	All kinds of biscuits according to their commercial name, including wafers. Savoury biscuits were excluded
Bread	Bread (soft) and similar products made with yeast
Breakfast cereals	Flakes, muesli, granola, extruded, ready-to-eat cereals
Cereal cakes/crackers	Cereal cakes and crackers with no yeast or gasifiers added
Fruit drinks	Drinks with a minimum of 5% fruit or juice (juices and smoothies are not included)
Milk/dairy drinks	All kinds of milk, flavoured milk shakes and milk with other ingredients
Milk substitutes	Vegetable drinks made of soya, oat, rice, coco, nuts, tigernut, canary seed or any other vegetable ingredient except tomato
Plant-based meat analogues	Any product made to resemble meat, made with plant-based ingredients
Toasted bread and similar	Toasted bread and similar products made with yeast (low water content)
Yogurt/dairy dessert substitutes	Fermented products and desserts made of plant-based drinks
Yogurts/fermented milk	Yogurts, milk fermented with alternative bacteria (dairy desserts are not included)

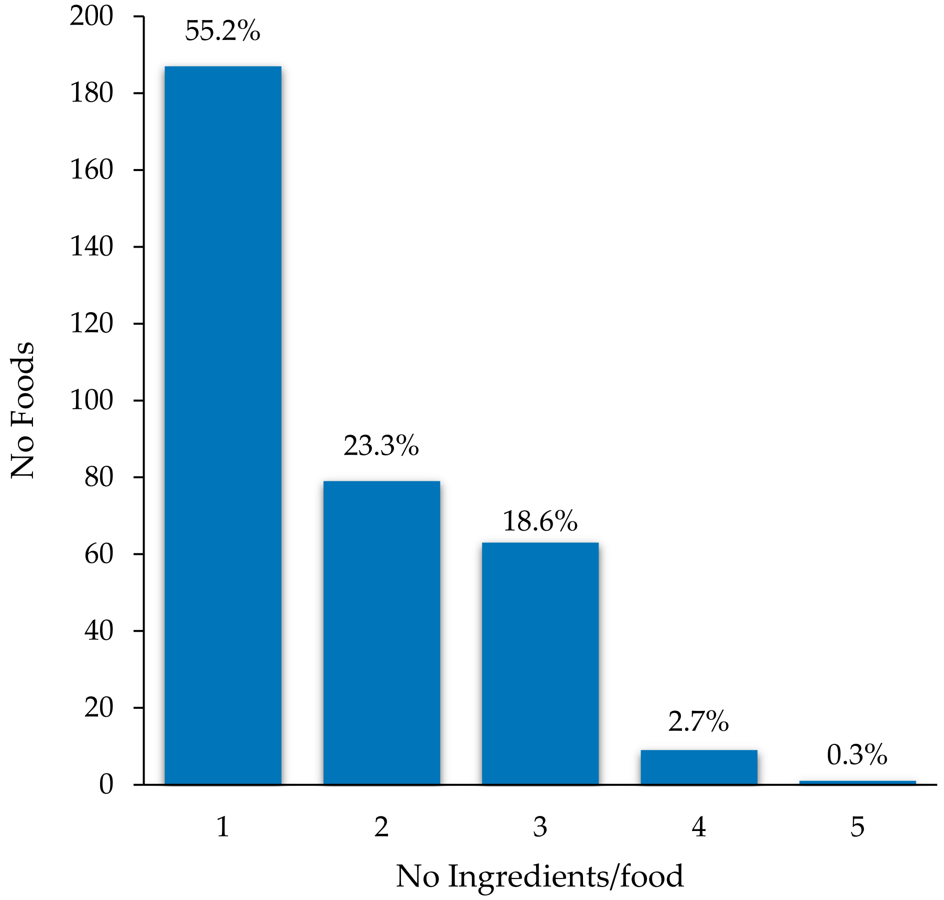

Figure S1. Presence of protein specific ingredients per food.

Table S2. Protein ingredients added to fortified foods with protein claims (PC).

**Proteins**	**Foods with PC**	
	**No**	**(%)**
**Animal proteins**	145	25.9
Bovine collagen peptides	6	1.1
Egg albumin	1	0.2
Egg white	16	2.9
Gelatine	10	1.8
Hydrolysed Pork Collagen Protein	22	3.9
Milk protein	105	18.7
Whey protein	44	7.8
**Plant proteins**	234	41.7
Almond protein	3	0.5
Corn protein	5	0.9
Fava bean and bean protein	8	1.4
Gluten *	106	18.9
Lupin protein	1	0.2
Pumpkin seed protein	1	0.2
Red lentil protein	1	0.1
Pea Protein	77	13.7
Potato protein	6	1.1
Rice protein	13	2.3
Sorghum protein	3	0.5
Soy protein	104	18.5
Sunflower seed protein	1	0.2
Wheat protein	38	6.8
**Others**	4	0.7
BCAAs (Leucine, Isoleucine, Valine)	1	0.2
Calcium caseinate	3	0.5

No: number of foods; PC: protein claims; %: percentage of the total foods with PC (both fortified and unfortified); *: gluten added to bread is not included here (see Material and methods).

Table S3. Number of foods with PC and protein ingredients added, by food type and ingredient.

Ingredients	Bars	Bread	Breakfast cereals	Cereal cakes/ crackers	Milk/dairy drinks	Milk substitutes	Plant-based meat analogues	Toasted bread and similar	Yogurt/dairy dessert substitutes	Yogurts/ fermented milk
Almond protein	0	0	2	0	0	0	0	0	1	0
BCAAs (Leucine, Isoleucine, Valine)	0	0	0	0	1	0	0	0	0	0
Bovine collagen peptides	6	0	0	0	0	0	0	0	0	0
Calcium caseinate	3	0	0	0	0	0	0	0	0	0
Corn protein	0	1	0	0	0	0	2	2	0	0
Egg albumin	0	0	0	0	0	0	1	0	0	0
Egg white	3	0	0	0	0	0	13	0	0	0
Fava bean and bean protein	0	0	0	0	0	0	8	0	0	0
Gelatine	2	0	0	0	0	0	0	0	0	8
Gluten	2	0	2	0	0	0	102	0	0	0
Hydrolysed pork collagen protein	22	0	0	0	0	0	0	0	0	0
Lupin protein	0	1	0	0	0	0	0	0	0	0
Milk protein	65	0	0	0	15	0	0	0	0	25
Pumpkin seed protein	1	0	0	0	0	0	0	0	0	0
Pea protein	13	2	2	0	0	3	56	1	0	0
Potato protein	0	0	0	0	0	0	6	0	0	0
Red lentil protein	0	0	0	0	0	0	1	0	0	0
Rice protein	4	1	3	0	0	0	2	3	0	0
Sorghum protein	0	1	0	0	0	0	0	2	0	0
Soy protein	29	6	1	1	0	0	67	0	0	0
Sunflower seed protein	0	0	0	1	0	0	0	0	0	0
Wheat protein	0	8	0	1	0	0	26	3	0	0
Whey protein	31	2	0	0	4	0	0	0	0	7

Table S4. Protein content in foods with protein claims (PC).

Food Types	Fortified	Protein (g)		
		n	Median (IR)	*p*-value
Bars	No	9	12.9 (9.3; 17)	< 0.001*
	Yes	86	30 (26.1; 36)	
Bread	No	28	10 (9.6; 12.2)	< 0.001*
	Yes	8	25.8 (23.5; 27.3)	
Milk/dairy drinks	No	13	5.2 (3.3; 8)	0.150
	Yes	18	7.9 (5.6; 9.7)	
Plant-based meat analogues	No	20	9.5 (7.8; 13.6)	< 0.001*
	Yes	173	16.3 (12; 19.6)	
Milk substitutes	No	35	ND	ND
	Yes	3	ND	
Yogurt/dairy dessert substitutes	No	29	ND	ND
	Yes	1	ND	
Yogurts/fermented milk	No	56	3.9 (2.9; 5.6)	< 0.01*
	Yes	39	6.2 (4.2; 7.9)	

n: Foods with data. IR: interquartile range. *: Statistically significant differences according to $p < 0.05$.
ND = not determined because of < 8 items/condition.

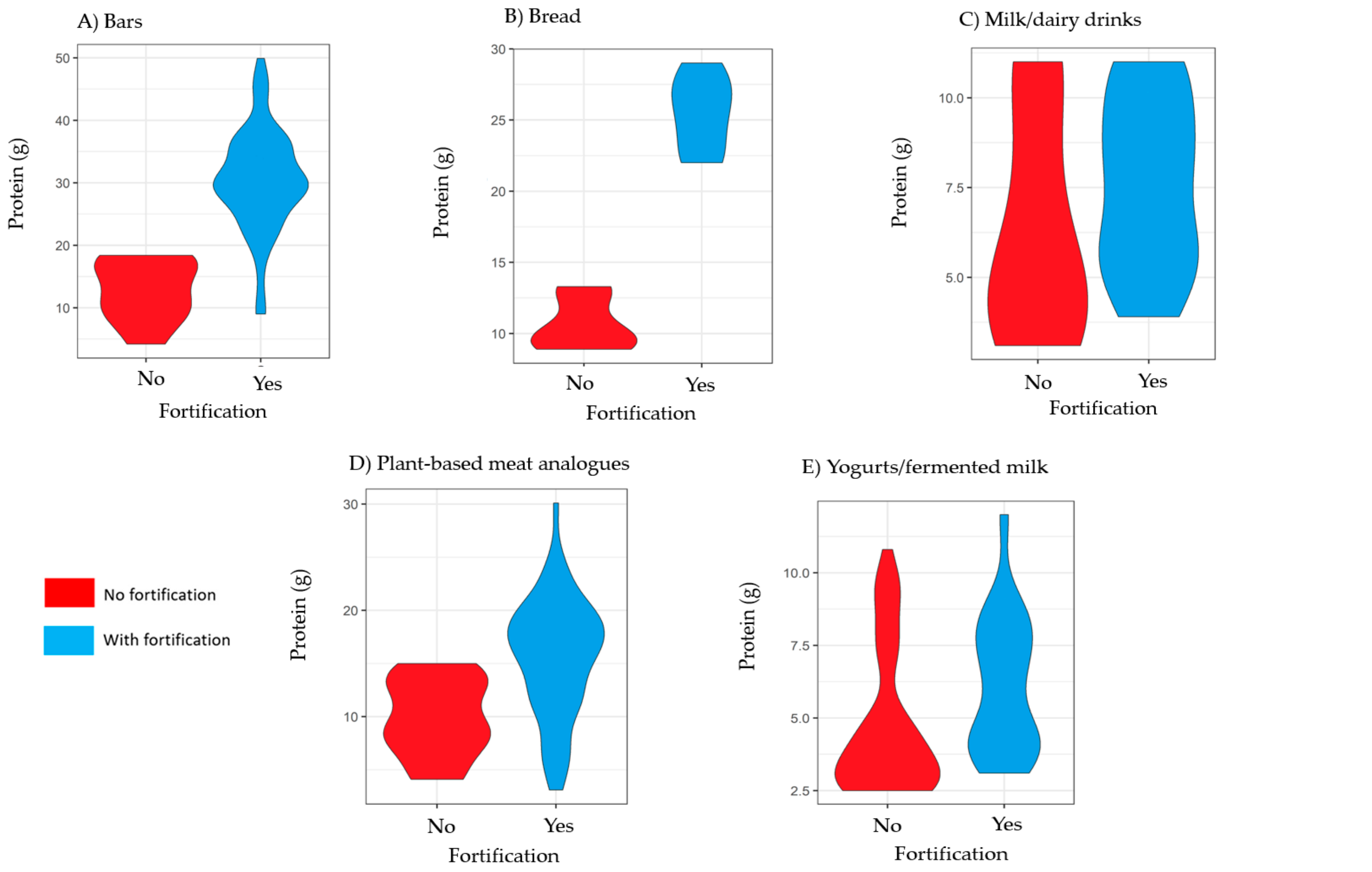

Figure S2. Protein content in foods with protein claims (PC). A) Bars; B) Bread; C) Milk/dairy drinks; D) Plant-based meat analogues; E) Yogurts/fermented milk.
